# Exploring targeted therapy in retinal vasculopathy with cerebral leukoencephalopathy: a case report and review of literature

**DOI:** 10.3389/fimmu.2025.1707532

**Published:** 2026-01-14

**Authors:** Patricia Tato-Moreno, Cristina Lavilla Olleros, Héctor Balastegui Martín, María Barrientos Guerrero, Anna Mensa-Vilaro, María Esther Durán-García, Paloma Sánchez-Mateos, Elena García-Martínez

**Affiliations:** 1 Immunology Department, Hospital General Universitario Gregorio Marañón, Madrid, Spain; 2 Instituto de Investigación Sanitaria Gregorio Marañón, Madrid, Spain; 3 Department of Internal Medicine, Hospital General Universitario Gregorio Marañón, Madrid, Spain; 4 Primary Immunodeficiencies Unit, Hospital Universitario y Politécnico La Fe, Valencia, Spain; 5 Immunology Department, Hospital Clínic of Barcelona, Barcelona, Spain; 6 Institut d’Investigacions Biomèdiques August Pi i Sunyer (IDIBAPS), Barcelona, Spain; 7 Pharmacy Department, Hospital General Universitario Gregorio Marañón, Madrid, Spain

**Keywords:** autoinflammation, case report, JAK-inhibitor, RVCL-S, targeted-therapy, TREX1, vasculopathy

## Abstract

Retinal vasculopathy with cerebral leukoencephalopathy and systemic manifestations (RVCL-S) is a rare autosomal dominant microvascular disorder caused by C-terminal truncating mutations in TREX1 gene, which impair protein localization and lead to multisystem involvement. We report a patient carrying the pathogenic TREX1 variant NM_033629.6:c.703dup (p.Val235fs), the most frequently described mutation in RVCL-S, whose clinical course was consistent with the classic phenotype but with simultaneous pulmonary granulomatous lesions compatible with sarcoidosis. Transcriptomic analysis in both the patient and his pre-asymptomatic daughter, who carries the same variant, revealed a similarly mild upregulation of inflammatory signaling pathways. Treatment with a Janus kinase inhibitor in the patient was followed by transient clinical stabilization before subsequent progression. This case expands the phenotypic spectrum of RVCL-S and underscores the importance of systematic immunological monitoring and clinical surveillance to support future development of timely strategies in asymptomatic carriers.

## Introduction

TREX1 gene encodes a cytosolic 3’-to-5’ DNA exonuclease that is expressed in most mammalian tissues and cell types (1). The N-terminal domain of the TREX1 protein is responsible for its catalytic activity and primary canonical function: degrading cytosolic single-stranded DNA and double-stranded DNA, thereby preventing self and foreign DNA accumulation and the activation of cytosolic DNA sensors, such as the cGAS-STING pathway. Additionally, TREX1 contains a C-terminal domain with a single-pass transmembrane motif that anchors the enzyme to the outer membrane of the nucleus that is linked to the endoplasmic reticulum (ER). This anchoring is essential for its cytosolic localization (2).

Mutations impairing TREX1’s nuclease activity lead to the accumulation of DNA and micronuclei in the cytosol, subsequently triggering a type I interferon (IFN) response via the cGAS-STING pathway (2). Such mutations are associated with autoinflammatory and autoimmune pathologies, including Aicardi-Goutières syndrome (AGS) type 1, systemic lupus erythematosus susceptibility and familial chilblain lupus. AGS is an early-onset encephalopathy that manifests in childhood and is characterized by neurological inflammation with calcification of white matter and the basal ganglia (3, 4). It is classified as a type I interferonopathy with both autosomal recessive and dominant inheritance pattern (5).

In contrast, disruption of the C-terminal domain results in cellular mislocalization of TREX1 protein, causing an even rarer disease with a distinct clinical presentation and autosomal dominant inheritance: retinal vasculopathy with cerebral leukoencephalopathy and systemic manifestations (RVCL-S). RVCL-S is an adult-onset vasculopathy that affects multiple organs, including the brain, kidney and retina. This condition is difficult to diagnose due to its low prevalence and currently has no effective treatment, resulting in a fatal outcome (6).

The striking differences in clinical presentation between these pathologies can be attributed to the underlying molecular defect (7). Interestingly, in some patients with RVCL-S, increased IFN signaling has also been observed, raising the question of how TREX1 is modulating IFN response and if this condition might also qualify as a type I interferonopathy. Additionally, treatment with Janus kinase (JAK) inhibitors have shown effectiveness in a patient with RVCL-S (8).

In this paper, we describe a patient with molecular diagnosis of RVCL-S who presented with atypical lung granulomas and elevated expression of inflammatory signaling pathways not associated with type I IFN. The patient has a daughter who carries the same mutation associated with RVCL-S. Although she is still asymptomatic, the question arises regarding the best monitoring and prevention strategies to follow in such patients.

## Case presentation

A 57-year-old Romanian man, previously healthy except for age-related conditions such as hypertension, as well as active smoking and chronic alcoholism, reported a two-year history of paraesthesias in the lower limbs with progressive muscle atrophy. In August 2022, his condition deteriorated significantly, with worsening limb weakness, pain, and functional limitation, eventually becoming incapacitating for normal ambulation. This was accompanied by noticeable changes in mood and cognitive status, prompting hospital admission.

**Figure 1:**
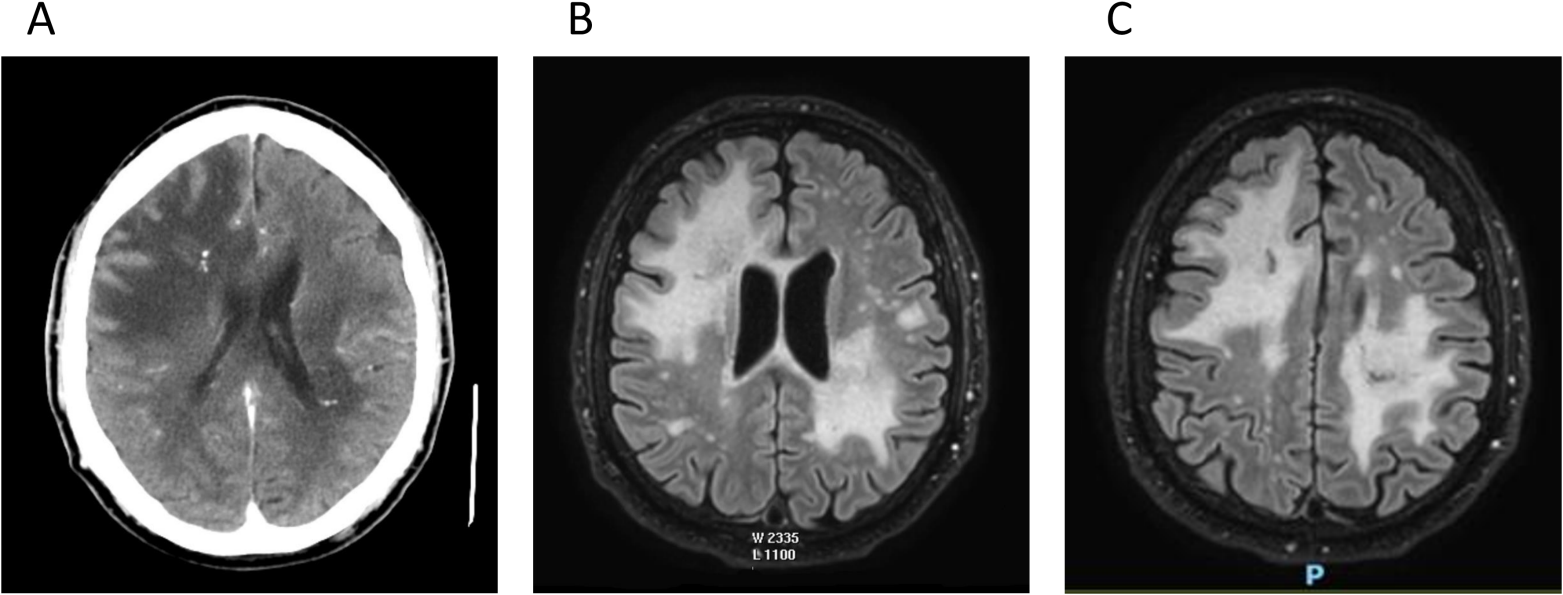
Computed tomography **(A)** and magnetic resonance imaging **(B, C)** reveal lesions affecting both the superficial and deep white matter in both cerebral hemispheres. The lesions exhibited faint ring enhancement and focal calcifications were notably present.

Brain magnetic resonance imaging (MRI) revealed lesions involving both the superficial and deep white matter, with extension through the corpus callosum and the left corticospinal tract (**Figure 1**). Some lesions had a pseudotumoral appearance and showed faint ring enhancement. A brain biopsy was performed to rule out neoplastic or infectious causes, revealing sterile necrotizing lesions. In the absence of apparent tumoral or infectious pathology, the findings were considered possibly ischemic in origin, potentially related to vasculitis, although this could not be definitively confirmed or excluded.

A month later, the patient presented with intestinal perforation complicated by secondary sepsis, which required admission to the intensive care unit. Intestinal biopsy findings suggested transmural ischemic necrosis with evidence of small and medium vessel neutrophilic vasculitis. Given the suspicion of polyarteritis nodosa-like vasculitis involving the gastrointestinal tract and nervous system, treatment was initiated with glucocorticoids and 500 mg of intravenous cyclophosphamide every two weeks.

**Figure 2:**
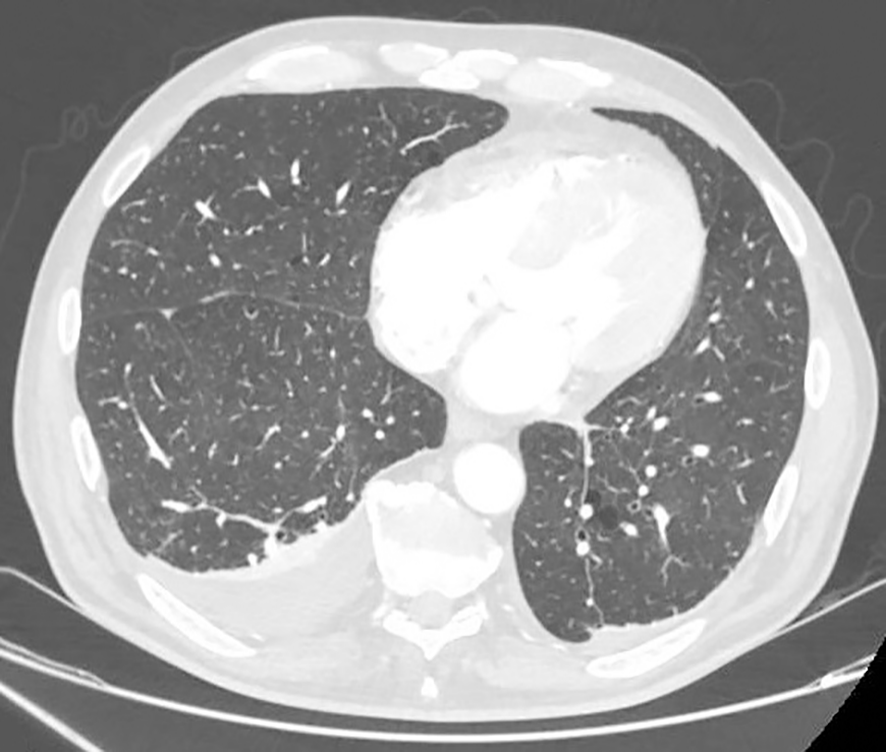
Computed tomography imaging revealed bilateral pleural effusions, with greater accumulation on the right lobe.

Initially, the patient progressed favorably; however, he was later readmitted with respiratory manifestations, including febrile episodes with profuse sweating and bilateral pleural effusion evident as a consolidated opacity on chest radiograph and confirmed by computed tomography (**Figure 2**). The pleural effusion was recurrent despite two weeks of oral levofloxacin 750 mg/24h. Biopsy findings suggested a granulomatous process in both pleura and hilar adenopathy, compatible with sarcoidosis, which was therefore considered a possible differential diagnosis, although the pattern of cerebral lesions was not typical of this condition. In view of a positive Mantoux and Quantiferon test indicating suspicion of tuberculosis, treatment was initiated for three months despite the absence of microbiological confirmation of active infection. Moreover, an ophthalmological assessment was requested, demonstrating the presence of retinal vasculitis with retinal white exudates.

With infectious etiology appearing to be excluded and given findings compatible with sarcoidosis with extrapulmonary involvement, treatment with subcutaneous methotrexate 15 mg weekly was started.

Regarding the family history, it is notable only for the patient’s father and paternal uncle, who both reportedly died due to brain tumors at around 40 years of age, according to family reports, although no confirmatory medical records are available.

Given the presence of an aggressive systemic inflammatory disease with pulmonary, digestive, ocular, and nervous system involvement, an extensive immunological workup was requested, including autoimmune screening, which yielded negative results, as well as an immunogenetic study.

## Genetic and transcriptomic analysis

Genomic DNA was isolated from peripheral blood and Next Generation Sequencing was performed using a clinical exome solution (4493 genes, Sophia Genetics). A panel of 304 genes associated with immune-mediated diseases were differentially analyzed (**Supplementary Table 1**).

**Figure 3:**
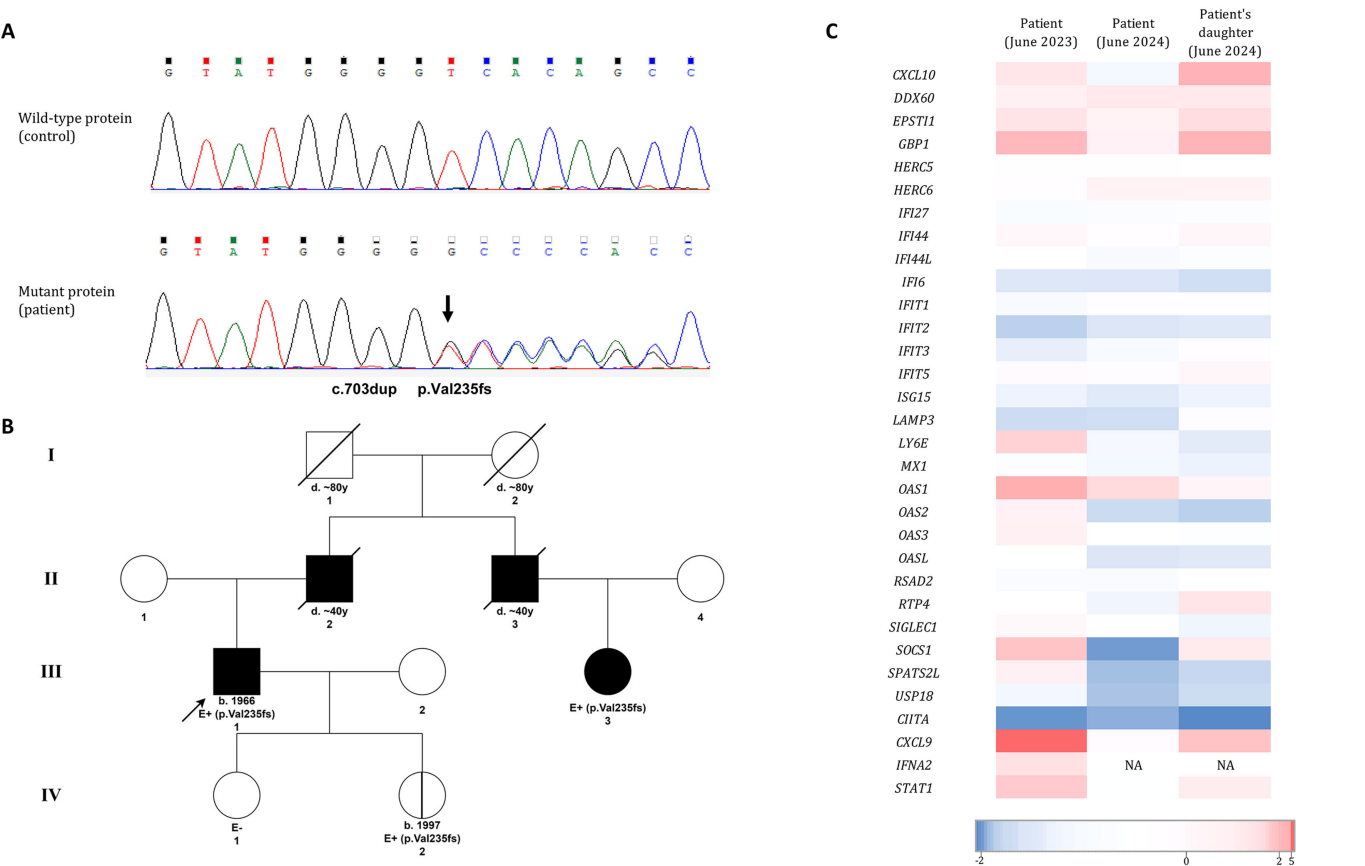
**(A)** Sanger sequencing results. The arrow indicates the nucleotide duplication NM_033629.6:c.703dup, which results in the frameshift variant p.Val235fs in the TREX1 protein. **(B)** Patient’s family pedigree. The arrow marks our patient (proband). Shaded boxes represent individuals diagnosed with RVCL-S. Box with vertical line (patient’s daughter, IV.2) represents presymptomatic carrier. “E” indicates the individuals that have been evaluated by genetic testing. E+ indicates a positive genetic test for the *TREX1* variant p.Val235fs. E- indicates a negative genetic test for the variant. **(C)** Heatmap of the individual gene z-scores in the patient before (June 2023) and after (June 2024) treatment with ruxolitinib and in the patient’s daughter (June 2024). NA, not assessable.

A heterozygous variant was found in *TREX1* gene (NM_033629.6:c.703dup, p.Val235fs), which was subsequently confirmed by Sanger sequencing (**Figure 3A**). This variant has extremely low frequency in population databases (gnomAD allele frequency = 0.0001239%; rs1553820434) and it has been extensively described as a pathogenic variant causing RVCL-S, being the most frequent variant identified in patients with this disease (9). The variant is located in the C-terminal domain of TREX1 protein and causes a shift in the reading frame of the coding sequence with the appearance of a premature stop codon (1). The resulting mRNA is predicted to escape nonsense-mediated mRNA decay mechanisms, producing a mutated protein truncated at the C-terminal domain (10).

Considering the information described thus far in our patient, and applying the criteria established by the American College of Medical Genetics and Genomics (ACMG) for variant interpretation (11), the variant was classified as pathogenic, supporting the molecular diagnosis of retinal vasculopathy with cerebral leukoencephalopathy and systemic manifestations (**Supplementary Table 2**).

Regarding the genetic segregation analysis of the variant within the family, samples were available only from the patient’s two daughters, one of whom was identified as an heterozygous carrier. The carrier daughter, a 27-year-old woman, remains asymptomatic to date, which aligns with previous reports indicating that individuals with this condition often remain symptom-free at this age. In addition, a first-degree cousin of our proband has recently been diagnosed with the disease, with identification of the same variant, which suggests that both the uncle and the father, who died due to brain tumors, suffered from RVCL-S (**Figure 3B**).

Evaluation of the IFN signature was performed through RNA extraction from peripheral blood. A total of 32 genes were analyzed using the NanoString nCounter Elements platform, including 28 type I IFN-response genes (IRGs) and 4 additional IFN-related genes. Z-scores were calculated for each gene based on a reference cohort of healthy controls. To assess IFN pathway activation, two global Z-scores were obtained by calculating the median of the individual Z-scores for two predefined IRG sets (28-gene and 6-gene panels). Additionally, a 3/25 gene expression ratio, which compares the expression of NF-κB/STAT1-regulated genes to those regulated by STAT1 alone, was calculated to explore potential involvement of the NF-κB pathway.

In the patient’s sample, neither of these global Z-scores exceeded the defined thresholds, indicating no evidence of a positive type I IFN signature. However, the 3/25 NF-κB-related expression ratio was slightly elevated at 0.20 (with 0.16 being the threshold for positivity), suggesting a modest involvement of the NF-κB pathway. A heatmap of individual z-scores is shown in **Figure 3C**. Numerical results of individual and global z-scores are shown in **Supplementary Table 3**. It must be considered that the patient was already undergoing immunosuppressive treatment with methotrexate at the time of the study.

Despite this, treatment with the JAK inhibitor ruxolitinib 5 mg/12h was initiated in November 2023, based on recent reports supporting its effectiveness in similar clinical contexts (8). The treatment was initially well tolerated and managed to control ocular (vascular retinopathy), systemic (microvascular renal disease, anemia, hypertension…) and neurological involvement. Six months after initiating treatment, the IFN signature was re-evaluated in the patient. The Z-score associated with the 28 IRG genes remained negative, and the 3/25 NF-κB-related ratio normalized to 0.12.

Additionally, the IFN signature was assessed for the first time in the patient’s asymptomatic carrier daughter. While her overall Z-score for IRG genes was negative, the NF-κB ratio was 0.20, equal to the value observed in her father before starting ruxolitinib treatment. Notably, some individual Z-scores for the expression of certain genes, such as *CXCL10* and *GPB1* (an IFN-inducible GTPase) were also slightly positive.

Given that the evaluation of the IFN signature is conducted using a transcriptomic approach that is highly susceptible to interference to treatments or presence of concomitant infections (including subclinical infections), additional sample collections would be considered in the future as part of the ongoing monitoring of the patient and his daughter.

**Figure 4:**
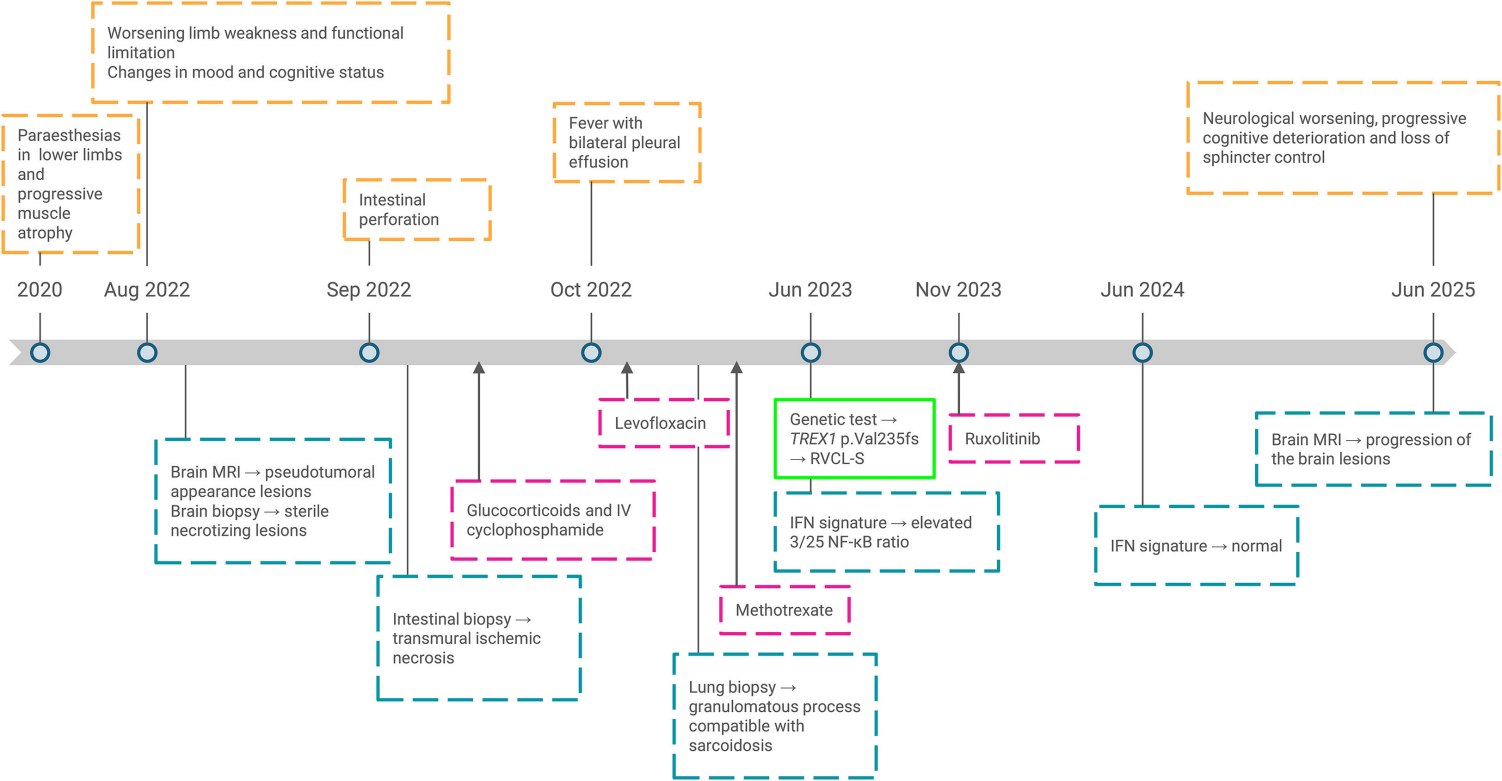
Clinical timeline of patient’s follow-up from the beginning of the symptoms until present. MRI, magnetic resonance imaging; IV, intravenous; RVCL-S, Retinal vasculopathy with cerebral leukoencephalopathy and systemic manifestations; IFN, interferon.

Finally, 18 months later, despite the treatment with ruxolitinib, the patient presented a neurological worsening with progressive cognitive deterioration and loss of sphincter control, typical of spinal cord dysfunction (**Figure 4**), which was confirmed by brain MRI showing progression of the brain lesions.

## Discussion

In this report, we present a case of retinal vasculopathy with cerebral leukoencephalopathy and systemic manifestations that follow the typical clinical course of the disease, with the addition of pulmonary lesions suggestive of sarcoidosis. Granulomatous-type lesions has not been described in the previously published TREX1 case reports, although it remains unclear whether there is a causal association or which underlying mechanisms typically associated with RVCL−S might be involved. This finding, together with the analysis of IFN-related markers, prompted us to question the potential contribution of inflammatory mechanisms to the clinical presentation in this patient. We describe the use of anti-JAK targeted therapy in our patient and its outcomes. The resulting temporary clinical stabilization, however, prevents establishing firm conclusions about the role of inflammation in the disease course.

The type of pathology associated with pathogenic variants in TREX1 depends on whether the nuclease activity or protein localization is affected (7). C-terminal truncating variants are linked to RVCL-S, which is consistent with our patient’s presentation, characterized by adult-onset multisystemic vasculopathy with progressive neurological and retinal involvement.

Patients with RVCL-S often do not develop clinical manifestations until the fourth or fifth decade of life, although early symptoms, such as Raynaud’s syndrome or visual dysfunction, may appear during this phase, which is often considered clinically asymptomatic. Later decades are marked by abrupt pathological progression, leading to premature death in all affected patients, typically within 5–10 years from the onset of symptoms (6, 8).

TREX1 lacking the transmembrane domain retains its exonuclease activity but is mislocalized within the cell. It remains unclear whether this mislocalization compromises its ability to prevent cytosolic DNA accumulation (1). Moreover, in contrast to AGS, the role of inflammation in RVCL-S is controversial. While local inflammation has been observed in brain lesions and some patients exhibit an elevated IFN signature (12), other studies show no significant differences in IFN-associated gene expression between RVCL-S patients and controls. Thus, it is uncertain whether inflammation contributes to the early mechanisms of pathogenesis or may arise in some patients as a consequence of the disease (7, 13).

One of the most recent hypotheses suggests that C-terminal truncated TREX1 proteins are able to enter the nucleus, where they contribute to chronic and progressive DNA damage and instability, leading to cytotoxicity and premature inflammation-mediated cell death. This aligns with the late age-related onset of symptoms observed in these patients and the resemblance of brain lesions to those seen in individuals with DNA damage induced by ionizing radiation or DNA repair deficiencies, such as ataxia-telangiectasia (13). In another study, preservation of the exonuclease domain was shown to be a critical determinant of disease, as the level of preserved activity correlated with the presence of typical RVCL-S features. The authors also demonstrated that truncated TREX1 proteins can induce nuclear DNA damage, particularly in endothelial cells, linking misdirected nuclease activity to the characteristic microvascular pathology (14).

Other authors propose that the underlying molecular defects in RVCL-S involve a dysregulation of the oligosaccharyltransferase complex, a multimeric enzyme structure in the ER-membrane that catalyzes N-linked protein glycosylation and whose activity appears to be modulated by the C-terminal domain of TREX1 when situated in ER-membrane. In the study by Hasan et al., the authors found that the C-terminal domain of TREX1 stabilizes the catalytic activity of the oligosaccharyltransferase complex, thereby preventing inflammation and the production of autoantibodies. Truncation of this C-terminal domain leads to enhanced hydrolysis of lipid-linked oligosaccharides, increasing the release of immunogenic free glycans that activate immune responses. This increased glycan hydrolysis reduces the available pool for N-glycosylation, resulting in the emergence of protein neo-epitopes and subsequent development of autoantibodies (7). In a mouse model heterozygous for the TREX1-V235fs variant, the mice exhibited moderated but significant elevated expression of IRG genes and presence of disease-associated autoantibodies. Among the IRG genes analyzed, CXCL10 has been proposed as one of the molecules involved in the associated systemic vascular dysfunction and endothelial damage, as it has previously been described as a chemokine capable of inhibiting angiogenesis and inducing the dissociation of newly formed vessels (7, 8).

Due to the limited understanding and lack of consensus regarding the pathogenic mechanisms of the disease, there are currently no well-defined or effective treatment regimens. Generally, the therapeutic approach involves close monitoring to allow for early intervention through standard care for the most common clinical manifestations (15, 16). In addition, immunosuppressive therapies, predominantly corticosteroids, are usually attempted, though with inconsistent results, with some patients experiencing symptom alleviation while others show no meaningful response. These differences may reflect underlying variability in the degree of autoimmune or autoinflammatory involvement across patients, as suggested by some authors (17). A new therapeutic intervention using the JAK inhibitor ruxolitinib has recently been described in a patient with the same *TREX1*-V235fs variant as our patient. The treatment resulted in the stabilization of visual and neurological deterioration symptoms, a reduction in peripheral blood *CXCL10* RNA levels (which were found elevated prior to treatment), and a significant regain of body weight (8).

In our patient, although IFN signature and individual CXCL10 Z-score were negative before treatment, possibly influenced by prior immunosuppressive therapy, treatment with ruxolitinib was well tolerated and was followed by stabilization of symptoms for eighteen months. Additionally, there was normalization of the 3/25 ratio score associated with NF-κB–mediated inflammation, which had been slightly elevated prior to treatment. Individual z-scores of other genes that had been elevated also normalized after therapy, including CXCL9 which, like CXCL10, is an IFN-inducible CXCR3 ligand with pro-inflammatory and vasculopathy-associated functions (18). However, after this period, the patient experienced clinical and radiological worsening, with progression of cerebral lesions. The limited duration of stabilization, the clinical complexity of the disease, and the multiple treatments often administered at the time of diagnosis make it difficult to determine to what extent inflammatory mechanisms represent a key component of disease pathogenesis. Consequently, it is not possible to establish a clear therapeutic benefit of ruxolitinib. In addition, cases of naturally slower disease progression and transient clinical stabilization without specific therapy have been described (19). Altogether, these factors prompt consideration of alternative therapeutic strategies, although currently available options remain limited.

In the asymptomatic carrier daughter, who is not receiving immunosuppressive treatment, the individual Z-score for CXCL10 expression in peripheral blood was slightly elevated, along with a subtle increase in the NF-κB ratio. Considering the cases reported to date, this condition appears to be a monogenic disorder with complete penetrance, which makes finding the optimal monitoring strategy for asymptomatic carriers during their early decades of life one of the major challenges moving forward. Moreover, it will be crucial to identify whether any early therapeutic interventions could delay the onset of symptoms and improve prognosis. In this context, targeted therapies capable of stabilizing disease course may represent promising options in selected asymptomatic carriers, particularly those with subclinical alterations. Reliable biomarkers and comprehensive immunological screening, including IFN signatures and transcriptomic analyses, may be useful to enable early identification of individuals at risk and to optimize therapeutic timing. However, the limited evidence currently available in this field complicates clinical decision-making, as illustrated by the daughter of our index case, who presents elevated IFN-related markers but for whom initiation of treatment remains under discussion among clinicians.

In addition to immune-derived biomarkers, other studies have explored indicators of neurological damage, such as serum and cerebrospinal fluid (CSF) neurofilament light chain (NfL) levels. In this analysis, CSF NfL levels were increased in pre-symptomatic individuals relative to controls, suggesting that CSF NfL could act as an early indicator of small vessel disease–related neural injury (20). Endothelial damage biomarkers have also been investigated as early indicators of vascular dysfunction. Serum von Willebrand factor antigen (VWF: Ag) was markedly elevated even in mutation carriers with minimal clinical manifestations compared with control (21), together with a pronounced imbalance between von Willebrand factor and its negative regulator ADAMTS13 (19). Increased endothelial accumulation of VWF: Ag was observed in brain tissue, suggesting that deeper characterization of this pathway may be useful for the development of targeted therapeutic strategies.

A recent clinical study has evaluated Crizanlizumab, a monoclonal antibody targeting P-selectin, in patients with RVCL-S. P-selectin plays a key role in leukocyte adhesion to the vascular endothelium, a process that contributes to microvascular occlusion and ischemia in various pathologies. The study demonstrated an acceptable safety profile for Crizanlizumab, stabilization of retinal symptoms as measured by perfusion indices, and showed the utility of the retina as a biomarker for systemic disease (22).

It should also be considered the importance of conducting an appropriate segregation study of the pathogenic variants within the family. In our patient, the initial evaluation of segregation was possible only in his two daughters. The patient’s father and paternal uncle had both passed away years earlier, in their forties, from what were described as brain tumors. Following further genetic evaluation within the extended family, a daughter of the patient’s paternal uncle, first-degree cousin of the index case, was also diagnosed with RVCL-S. This finding strongly suggests that she may have inherited the pathogenic variant from her father. Cerebral lesions in this condition are frequently initially misdiagnosed as tumors or demyelinating processes, particularly when the disease is not suspected. This may have been even more likely in cases that occurred decades ago, when diagnostic awareness and available tools were more limited. Although no definitive conclusion can be drawn, the available evidence supports the likelihood that both the patient’s father and uncle may also have carried the pathogenic variant.

Building on this possibility, these circumstances prompt consideration of whether the variant may have been inherited from a previous generation. However, both paternal grandparents died at an advanced age without any reported history suggestive of pathology potentially related to this condition, leading to the additional premise that one of them could have harbored a mosaicism involving the germline cells, thus allowing transmission of the variant to the next generation.

In line with this, the first case of *TREX1* mosaicism has been recently reported, diagnosed by the identification of a pathogenic variant in a skin biopsy and its absence in peripheral blood (6). The patient exhibited a remarkably mild phenotype, with RVCL-S manifestations limited to the retina and reaching advanced age without systemic involvement. However, germline transmission of the variant was confirmed, as all three of his children inherited the mutation and subsequently developed the full clinical spectrum of RVCL-S (6). The authors also made an initial approach of prime editor gene therapy in mice and human cells, suggesting that in the future, the conversion of patients from germinal to mosaic status could be another treatment alternative for this condition. Further refinement of this technology is still necessary, along with a better understanding of which cell lines would be most appropriate to target.

Understanding the precise pathogenic mechanisms and identifying the key molecular pathways involved in RVCL-S, including the role of inflammation, will be crucial for guiding the development of appropriate therapeutic strategies. Furthermore, assessing the effectiveness of targeted therapies that achieve clinical stabilization, together with proper immunological screening such as IFN signatures and other biomarkers of neurological and endothelial damage that correlate with treatment response, will be essential to establish reliable monitoring protocols and explore potential preventive interventions in asymptomatic carriers of the disease. Also, given the low prevalence and challenging nature of this disease, raising awareness and promoting collaboration between centers and researchers is essential. In this context, active participation in established initiatives and networks is of critical importance (23, 24).

## Data Availability

The original contributions presented in the study are included in the article/**Supplementary Material**. Further inquiries can be directed to the corresponding author.
